# Mobile Health in Oncology: A Patient Survey About App-Assisted Cancer Care

**DOI:** 10.2196/mhealth.7689

**Published:** 2017-06-14

**Authors:** Kerstin Anne Kessel, Marco ME Vogel, Carmen Kessel, Henning Bier, Tilo Biedermann, Helmut Friess, Peter Herschbach, Rüdiger von Eisenhart-Rothe, Bernhard Meyer, Marion Kiechle, Ulrich Keller, Christian Peschel, Roland M Schmid, Stephanie E Combs

**Affiliations:** ^1^ Klinikum rechts der Isar Department of Radiation Oncology Technical University of Munich (TUM) Munich Germany; ^2^ Institute for Innovative Radiotherapy (iRT) Department of Radiation Sciences (DRS) Helmholtz Zentrum München Neuherberg Germany; ^3^ Onkologisches Zentrum im RHCCC am Klinikum rechts der Isar Technical University of Munich (TUM) Munich Germany; ^4^ Klinikum rechts der Isar Department of Otorhinolaryngology Technical University of Munich (TUM) Munich Germany; ^5^ Klinikum rechts der Isar Department of Dermatology and Allergy Biederstein Technical University of Munich (TUM) Munich Germany; ^6^ Klinikum rechts der Isar Department of Surgery Technical University of Munich (TUM) Munich Germany; ^7^ Roman Herzog Comprehensive Cancer Center (RHCCC) Department of Psychosomatic Medicine and Psychotherapy Technical University of Munich (TUM) Munich Germany; ^8^ Klinikum rechts der Isar Department of Orthopedic Surgery Technical University of Munich (TUM) Munich Germany; ^9^ Klinikum rechts der Isar Department of Neurosurgery Technical University of Munich (TUM) Munich Germany; ^10^ Klinikum rechts der Isar Department of Gynecology and Obstetrics Technical University of Munich (TUM) Munich Germany; ^11^ Klinikum rechts der Isar 3rd Department of Internal Medicine Technical University of Munich (TUM) Munich Germany; ^12^ Klinikum rechts der Isar 2nd Department of Internal Medicine Technical University of Munich (TUM) Munich Germany

**Keywords:** clinical oncology, surveys and questionnaires, mobile apps, mHealth, eHealth

## Abstract

**Background:**

In the last decade, the health care sector has been enriched by numerous innovations such as apps and connected devices that assist users in weight reduction and diabetes management. However, only a few native apps in the oncological context exist, which support patients during treatment and aftercare.

**Objective:**

The objective of this study was to analyze patients’ acceptance regarding app use and to investigate the functions of an oncological app that are most required, and the primary reasons for patients to refuse app-assisted cancer care.

**Methods:**

We designed and conducted a survey with 23 questions, inquiring patients about their technical knowledge and equipment, as well as the possible advantages and disadvantages, data transfer, and general functionality of an app.

**Results:**

A total of 375 patients participated; the participation rate was 60.7% (375/618). Gender distribution was about 3:4 (female:male) with a median age of 59 years (range 18-92 years). Whereas 69.6% (261/375) of patients used mobile devices, 16.3% (61/375) did not own one, and 9.1% (34/375) only used a personal computer (PC). About half of the patients rated their usability skills as very good and good (18.9% 71/375; 35.2% 132/375), 23.5% (88/375) described their skills as intermediate, and 14.4% (54/375) as bad. Of all patients, 182 (48.5%, 182/375) were willing to send data to their treating clinic via an app, that is, to a server (61.0% 111/182) or as email (33.5%, 61/182). About two-thirds (68.7%, 125/182) believed that additional and regularly sent data would be an ideal complement to the standard follow-up procedure. Additionally, 86.8% (158/182) wished to be contacted by a physician when entered data showed irregularities. Because of lack of skills (34.4%, 56/163), concerns about the use of data (35.0%, 57/163), lack of capable devices (25.8%, 42/163), and the wish for personal contact with the treating physician (47.2%, 77/163), a total of 163 (43.5%, 163/375) patients refused to use an app. Pearson correlation showed a significant but mild relationship between age and app use (*P*=.03, *r*=−.12), favoring younger age; male gender correlated as well (*P*=.04; *r*=−.11).

**Conclusions:**

The results show that the introduction of mobile apps needs to follow different strategies depending on the patients’ attitude. Age and gender seem to be the strongest predictive factors. For oncology patients, our survey showed that about half of the patients were willing to send data via an app supporting their treatment. In the future, clinical data such as quality of life and treatment satisfaction recorded by mobile health (mHealth) devices could be used to evaluate and improve therapy workflow. Furthermore, apps could support classical visits, document adverse effects, and remind patients of treatment dates or drug intake.

## Introduction

In the last decade, apps for mobile phones and tablets changed our life completely. Since the introduction of iOS (Apple Inc., USA) in 2008, apps are ubiquitous, and more than 5 million apps are available in the leading app stores [1]. Many of these support us in our everyday lives and ensure time savings or entertainment: the possibilities are huge and range from simple weather apps to complex three-dimensional games. Also, the health care sector has been enriched by numerous new innovations such as apps for weight reduction, depression, or diabetes [2-4]. Wearables and devices such as fitness trackers, blood pressure monitors, blood glucose meters, and personal scale gears are popular and convey the impression of high acceptance for collecting medical data. The World Health Organization (WHO) defines all these tools under the labels electronic heath (eHealth) and mobile health (mHealth) [5].

Although the IT world states that the era of apps has already passed, to date, only a few native apps in oncological context exist, which support patients during treatment and aftercare, and at the same time enable data analyses and feedback strategies. Not only in oncology, but in general, health care apps often lack standardized validation regarding benefits, acceptance, costs, and risks [6]. Brouard et al [7] evaluated 117 apps for oncological information and treatment monitoring. The validation of those apps was poor (27.4%). A work by Collado-Borrell et al [8] pointed out a lack of professional involvement during development and validation of 166 apps for cancer patients. Only 48.8% were developed by health care organizations.

Recently, a randomized clinical trial by Denis et al [9] investigated the outcome of lung cancer patients and showed a significantly better survival for patients (median overall survival 19 vs 12 months) using a Web-based tool for periodical documentation of symptoms and side effects during follow-up. Earlier works of the research group showed higher compliance, better communication, and 5-week earlier detection of relapse [10].

There is an ongoing debate on patients’ and health care professionals’ (HCPs) opinion on app technology and telemedicine [11]. A recent survey of 108 HCPs could show a great acceptance (84.3%) of app-assisted treatment [12]. The digital medicine is unstoppable and patient empowerment plays a new and growing role in disease management.

During the certification process of our Oncology Center (Onkologisches Zentrum [OZ] am RHCCC am MRI TU Munich [TUM]), we analyzed patients’ acceptance regarding oncological apps. The aim of this study was to evaluate their concerns and requests. We investigated which functions are most required and what are the primary reasons for patients to refuse app-assisted cancer care.

## Methods

We designed a patient questionnaire with 23 questions (Q1-Q23), which included sociodemographic details and patients’ general opinion on oncological apps. Furthermore, inquiries were made on technical knowledge and equipment, possible advantages and disadvantages, data transfer, and general functionality. The survey was designed by experienced oncologists and medical computer scientists. Before initiation, the questionnaire was tested with 15 patients to optimize format and wording. Minor changes were made to provide a better patient-friendly understanding of the content of each question.

We used either multiple-choice questions with single (Q1, Q8-10, Q14, Q15-20) or multiple answers (Q4-6, Q9.1, Q9.2, Q12, Q13, Q21), free-text questions (Q1, Q2, Q3, Q23), or matrix/rating-scale questions (Q7, Q11, Q22). Rating scales were designed with even answers to avoid a central tendency bias. Q9 was developed as a polar question with branching logic with either answer “yes” (followed by Q9.1) or answer “no” (followed by Q9.2). Foreign words and technical terms were explained in a footnote where necessary (see Multimedia Appendices 1 and 2).

The evaluation was based primarily following the criteria of the Deutsche Krebsgesellschaft (DKG) for the certification of oncological centers in Germany. The survey was performed within the Oncology Center, Munich (Onkologisches Zentrum [OZ] am RHCCC am MRI TU Munich [TUM]) in the following units: dermatooncology (DERMA), breast center and gynecology (GYN), head-and-neck tumor center (HAN), hematooncology (HEM), neurooncology (NEURO), orthopedic surgery (ORTHO), radiation oncology (RADONC), and abdominal surgery (SUR). According to the expected average patient cases per month, we distributed a total of 750 questionnaires (Table 1).

The survey was conducted for 3 months from May to July 2016. Participation was voluntary and anonymous; hence, no written consent was required by the patient. Inclusion criteria for participation were as follows: age older than 18 years, German-speaking, and physical and mental ability to fill out a structured questionnaire. Research assistants collected the anonymized data in the institutional database. The Ethics Committee of the Technical University of Munich (TUM) approved the nature and content of the study with the project number 18/16 S.

Statistical calculations were performed using SPSS statistics version 23 (IBM Corp) in a primarily descriptive way. Bivariate Pearson correlations (2-tailed) were calculated for the relationship between app use and variables, which included gender, age group, and technical skills. *P*<.05 was considered as statistically significant.

## Results

Of all 750 distributed questionnaires, 375 were filled out and returned, whereas 132 were not used. This results in a participation rate of 60.7% (375/618). Gender distribution in the whole cohort was about 3:4 (female:male), with a median age of 59 years (range 18-92 years; Table 1; Q1, Q2).

Patients received the following therapies within the oncology center (Q4, multiple answers were possible): 44.3% (166/375) radiotherapy, 42.4% (159/375) chemotherapy, and 62.9% (236/375) surgery. Of all patients, 69.6% (261/375) owned a mobile device (mobile phone: 65.1%; tablet: 33.9%), whereas 16.3% (61/375) had no device, and 9.1% (34/375) only used a standard PC or notebook (Q5). Android (138/261, 52.9%) was the most commonly used mobile operating system (OS) (Q6), followed by iOS (97/261, 37.2%), Windows Mobile (31/261, 11.9%), and BlackBerry OS (2/261, 0.8%). About half of the patients rated their own usability skills (Q7) as very good (71/375, 18.9%) and good (132/375, 35.2%), whereas 23.5% (88/375) and 14.4% (54/375) described their usability skills as intermediate and poor, respectively.

**Figure 1:**
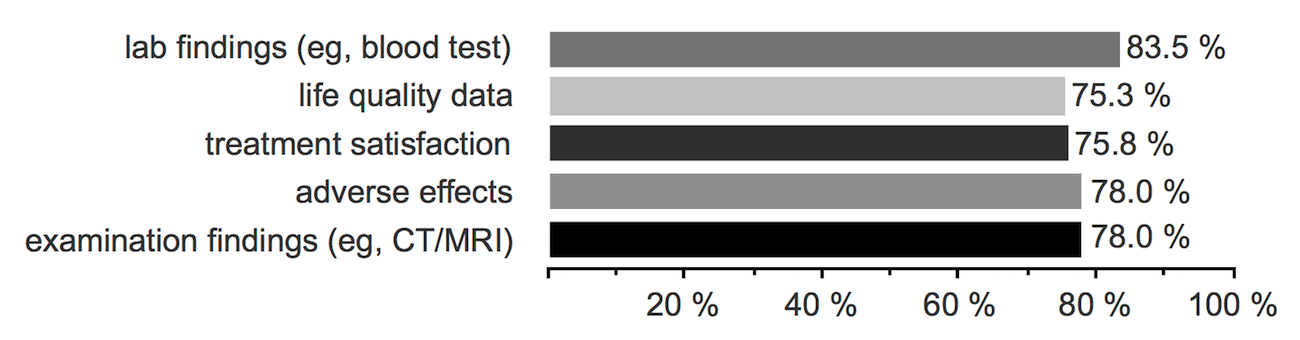
Q9.1: Which data are you willing to transfer? (n=182).

**Figure 2:**
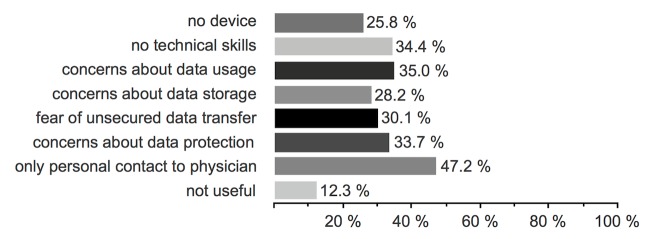
Q9.2: Why would you not send data via an app to your treating clinic? (n=163).

Of all patients, 48.5% (182/375) were willing to send medical data via an app to their treating clinic (Q9). While Figure 1 shows data types that patients are willing to send, Figure 2 lists the patients’ concerns and reasons to refuse sending data (43.5%, 163/375). When the health insurance offered a cashback or bonus when using the app as a supporting medical health tool (Q10), 36.3% (136/375) used it; however, 48.8% (183/375) were not influenced by that. Six patients (1.6%, 6/375) who previously stated they would not transfer data via an app changed their mind and used an app when financial compensation was offered (eg, by the health insurance).

**Table 1:** Patient distribution according to the participating oncological units.

Unit	Questionnaires distributed	Patients, n	Not used	Return rate	Gender				Median age (range) in years
					Female		Male		
All	750	375	132	60.7% (375/618)	169	45.1%	206	54.9%	59 (18-92)
DERMA^b^	50	36		72.0% (36/50	22	61.1%	14	38.9%	56 (18-81)
GYN^c^	50	6		12.0% (6/50)	6	100.0%	0	0.0%	59 (26-76)
HAN^d^	100	36	3	37.1% (36/97)	14	38.9%	31	86.1%	59 (38-85)
HEM^e^	50	42		84.0% (42/50)	19	45.2%	23	54.8%	63 (31-78)
NEURO^f^	150	45	77	61.6% (45/73)	20	44.4%	25	55.6%	54 (21-78)
ORTHO^g^	100	50	12	56.8% (50/88)	26	52.0%	24	48.0%	60 (18-92)
RADONC^h^	200	118	40	73.8% (118/160)	55	46.6%	63	53.4%	58 (18-81)
SUR^i^	50	42		84.0% (42/50)	16	38.1%	26	61.9%	62 (30-82)

^a^DERMA: dermatooncology.

^b^GYN: breast center/gynecology.

^c^HAN: head-and-neck tumor center.

^d^HEM: hematooncology.

^e^NEURO: neurooncology.

^f^ORTHO: orthopedic surgery.

^g^RADONC: radiation oncology.

^h^SUR: abdominal surgery.

**Figure 3:**
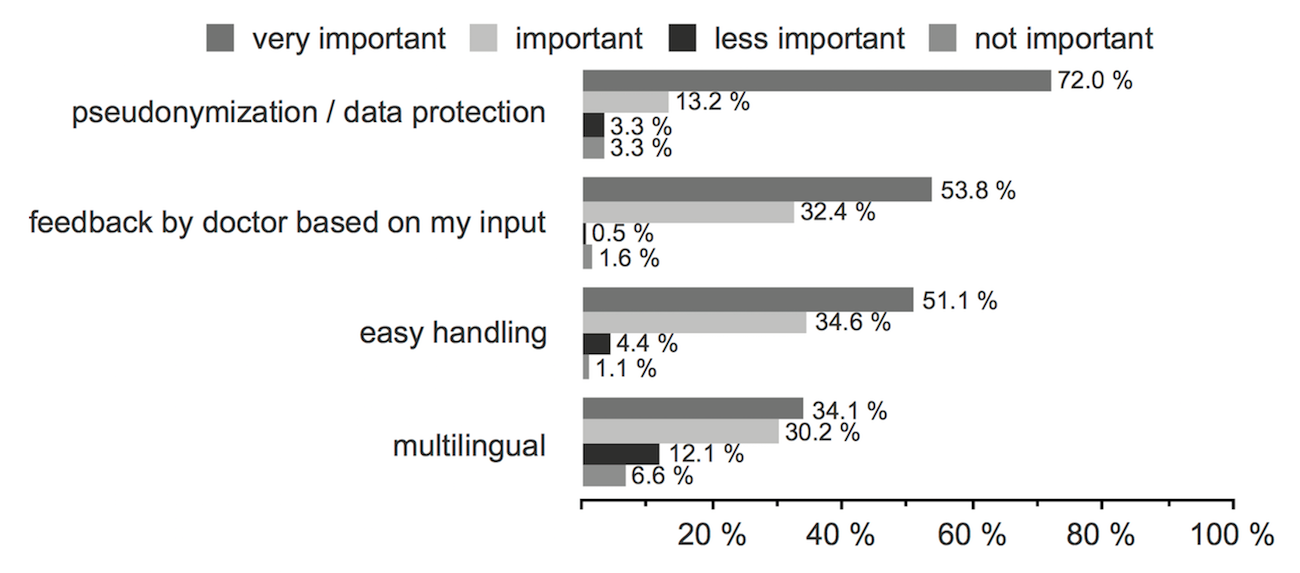
Q11: What would be important to you when considering using an app? (n=182).

The questions Q11-Q23 were only answered by patients who indicated they would use an app with secure data transfer (48.5%, 182/375). The most important characteristics of an app should be pseudonymization, data protection, as well as feedback by a physician based on the patients’ input (Figure 3, Q11). The patients agreed to the following data transfer options: data sent via the Internet to a server (61.0%, 111/182), via a cloud-based solution (11.0%, 20/182), via email (33.5%, 61/182), only on-site and locally in the clinic (19.2%, 35/182), or, for some the mode of transfer was irrelevant (10.4%, 19/182; Q12). Data entry was done at least every month (29.1%, 53/182) or every 3 months (26.4%, 48/182), in accordance to the follow-up appointments (26.4%, 48/182), and independently when necessary (17.6%, 32/182) were also favored options (Q13). The time required for data entry (eg, symptoms or current side effects) should not exceed 15 minutes (72.0%, 131/182; Q14). Additionally, 89.6% (163/182) agreed to the further use of their sent data for scientific evaluations (Q16).

About two-thirds (68.7%, 125/182) believed additional and regularly sent data would be an ideal complement to the standard follow-up procedure (Q19). About 86.8% (158/182) wished to be contacted by a physician when entered data showed irregularities (Q20).

**Figure 4:**
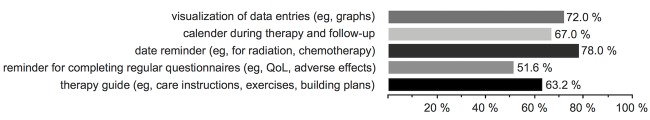
Q21: Figure listing desired additional functions (n=182).

Of all, 10.4% (19/182) also use other eHealth apps such as running apps or tracking apps for blood sugar, heart rate, or weight tracking (Q18); 10.4% (19/182) use eHealth devices such as step counters or heart rate monitors (Q17). Additional functions, apart from symptom tracking (Figure 4), were favored by 73.6% (134/182); in contrast, 15.9% (29/182) liked a simple clean app.

Furthermore, we also compared app use by age group (18-39 years; ≥40 years) and gender. Pearson correlation showed a significant but mild relationship between age and app use (*P*=.03, *r*=−.12) favoring younger age; technical skills (very good or good vs intermediate or bad) showed the same tendencies (*P* ≤ .001, *r*=−.28). Male gender and app use correlated as well with *P*=.04 (*r*=−.11).

## Discussion

Conducted at a large, university-based oncological center, our survey revealed that about half of the patients were willing to send data via an app supporting their oncological treatment and follow-up, whereas the patients’ refusal to use an app was primarily due to their fear of subsequent data use, lack of technical understanding, and data security reasons. The results showed that younger patients had a higher acceptance of these tools (*P*=.03 *, r*=−.12), as did male patients (*P*=.04, *r*=−.11). There was also a mild correlation (*P* ≤.001, *r*=−.28) between patients’ technical skills and their preference for apps. Thus, the introduction of mobile apps might need to follow different strategies depending on the patients’ attitude. Moreover, age and gender might be the strongest predictive factors. Younger patients show a greater inclination toward using an app. Older patients generally describe themselves as not highly skilled in the utilization of mobile phones and other mobile devices. The reluctance of female patients might be attributable to the general technical affinity of men. In contrast, a feasibility study about the use of mobile devices collecting patient-reported symptoms during radiotherapy by Falchook et al [13] showed no influence between any patient characteristic and reporting compliance; however, the cohort was relatively small with only 21 patients participating.

The possibilities for such apps are numerous. Clinical data could be used to evaluate and improve the departments’ therapy workflow. The integration of quickly available digital information into daily clinical workflow seems promising. If patients are well trained, they can give input on their health status and other information by themselves without any dependence on the capacity of a physician’s assistant, study nurse, or other. Moreover, information can be collected in real-time, which potentially bears high risks but also facilitates opportunities for fast response in situations of medical need. Furthermore, this type of data acquisition (eg, for blood counts and information on side effects) could be implemented in clinical studies. The limitations of our results are the relatively old patient cohort (median age 59 years) and the particular setting in oncology. The results may not apply to the general patient in other treatment situations.

However, the use of wearables and apps in the health care sector will grow strongly [2]. Chen et al [14] questioned 101 people using health care apps and 77% stated that they are willing to share their data for research. In our study on understanding the attitude of oncological patients toward app use, 48.5% (182/375) agreed to send personal and health data and make themselves available for further analyses. The expected profits in the areas of prevention, diagnostics, and therapy, as well as the increasing cost pressure for hospitals and health insurance will push mHealth innovations and drive the mobile transformation of all sorts of processes.

The compliance to use apps is high in various domains. A current health app revolution can be observed, which is exploited by many non-expert developers. Apps for oncological patients must be developed carefully by keeping in mind that the recipients are very vulnerable, as they mostly have to fight with quick relapse and bad prognosis and will use everything to improve their outcome. Cancer patients are always interested in doing everything possible to have a positive influence on their respective disease. Oncological apps could strengthen the self-care and allow close follow-up. However, a standardized validation process must be implemented for medical apps to guarantee safety for the patient [8,15,16]. Further prospective clinical trials, such as the study by Denis et al [10] on lung cancer, which proved a positive influence of apps during follow-up on survival, would be necessary to demonstrate their respective benefit for the patient before these are deployed to the public.

Young people grow up with apps in all life situations. The constant mobile availability of information is, therefore, self-evident to them. This generation will continue to drive the development and use of mobile apps also in the medical field and ensure that they ultimately determine the digital health care. This revolution will change the way physicians work and the role of data protection and its meaning for the patient [17].

Clinical data, such as quality of life and treatment satisfaction, recorded by mHealth devices could be used to evaluate and improve therapy workflow in the future, apps could support classical visits and document side effects, or remind patients of treatment dates or drug intake. The advantages could be equally beneficial for professionals and patients. Though mobile phones and other mobile devices will certainly not replace personal contact with a physician, these will serve as a digital assistant in diagnosis, therapy, and follow-up.

## Appendix

Multimedia Appendix 1 Original questionnaire (German).

Multimedia Appendix 2 Translated questionnaire (English).

## Abbreviations

DKG: Deutsche Krebsgesellschaft
eHealth: electronic health
HCPs: health care professionals
mHealth: mobile health
OS: operating system
PC: personal computer
TUM: Technical University of Munich
WHO: World Health Organization
