# Supplementary material for: Mobile Health in Oncology: A Patient Survey About App-Assisted Cancer Care
Source: JMIR Mhealth Uhealth. 2017 Jun 14;5(6):e81. doi: 10.2196/mhealth.7689 (PMC5489709; doi:10.2196/mhealth.7689)
Supplement: Multimedia Appendix 1 [file mhealth_v5i6e81_app1.pdf]

PATIENTEN - FRAGEBOGEN

Klinikum rechts der Isar

Technische Universität München

Liebe Patientin, lieber Patient,

Smartphone-*Apps* finden immer mehr Anwendung und unterstützen uns im Alltag. Auch in der medizinischen Versorgung kommen vermehrt elektronische / mobile Geräte zum Einsatz – allgemein als *eHealth* bezeichnet.

Gerne möchten wir herausfinden, wie diese Applikationen sinnvoll in unserer Klinik zu Ihrem Vorteil eingesetzt werden können. Hierfür benötigen wir Ihre Hilfe! Wir würden uns daher freuen, wenn Sie sich einige Minuten Zeit nehmen, um unseren Fragebogen zu einer Gesundheits-*App* in der Onkologie für Smartphones und Tablets im Patientenalltag zu beantworten. So können Sie uns bei der Gestaltung der App unterstützen.

Bitte geben Sie den ausgefüllten Fragebogen an der Anmeldung ab oder werfen Sie ihn in die vorgesehenen Briefkästen. Alle Angaben werden natürlich streng vertraulich behandelt.

Wir danken Ihnen sehr für Ihre Mithilfe!
Mit den besten Wünschen für Ihre Genesung,

Ihre

Stephanie E. Combs

1. Allgemeine Informationen:

Geschlecht
☐ weiblich
☐ männlich

Alter
____

Muttersprache
____

2. In welcher Abteilung werden Sie am MRI betreut?

____

3. Wegen welcher Tumor-Erkrankung sind Sie in Behandlung?

____

4. Welche Therapien erhalten Sie bzw. haben Sie in der Vergangenheit erhalten? (Mehrfachauswahl möglich)

☐ Strahlentherapie ☐ Chemotherapie ☐ Operation

5. Welche mobilen Geräte stehen Ihnen zur Verfügung? (Mehrfachauswahl möglich)

☐ Smartphone ☐ Tablet ☐ PC/Notebook ☐ keines

6. Welches Betriebssystem hat Ihr Smartphone/Tablet? (Mehrfachauswahl möglich)

☐ Android ☐ iOS (Apple) ☐ WindowsMobile ☐ BlackberryOS

☐ Andere: ____________________ ☐ weiß ich nicht

7. Wie sicher/routiniert fühlen Sie sich im Umgang mit mobilen Geräten?

☐ sehr gut ☐ gut ☐ mittelmäßig ☐ schlecht

8. Benutzen Sie Apps im Alltag?

☐ ja ☐ nein

Unter anderem die folgenden: ____

9. Würden Sie eine mobile Gesundheits-App befürworten/benutzen und uns Daten zum Verlauf Ihrer Erkrankung per App auf einem sicheren Weg übermitteln?

☐ ja ☐ nein

9.1. Wenn **ja**, welche Daten bzw. Informationen würden Sie an uns übermitteln? (Mehrfachauswahl möglich)

☐ Laborwerte (Blutwerte z.B. PSA-Wert)
☐ Daten zur Zufriedenheit / Unzufriedenheit mit der Behandlung
☐ Daten zu Lebensqualität und Wohlbefinden
☐ Untersuchungsergebnisse (z.B. eines CT/MRT)
☐ Daten über subjektive Nebenwirkungen / Schmerzen, die mit den Therapien verbunden sind

☐ Sonstiges: ____

9.2. Wenn **nein**, warum möchten Sie keine Daten übermitteln? (Mehrfachauswahl möglich)

☐ Ich habe keine passenden Endgeräte.
☐ Ich kenne mich nicht mit der Technik aus.
☐ Ich weiß nicht, was mit den Daten passiert.
☐ Ich weiß nicht, wo die Daten gespeichert werden.
☐ Ich befürchte keine gesicherte Datenübertragung.
☐ Datenschutzgründe (Ich möchte keine Übertragung von nicht anonymisierten[1] Daten.)
☐ Ich möchte ausschließlich persönlichen Kontakt zum Arzt.
☐ Ich halte es nicht für sinnvoll.

☐ Andere Gründe: ____

10. Würden Sie die App benutzen, wenn es dafür Bonusleistungen von Ihrer Krankenkasse gäbe?

☐ ja ☐ nein

Alle folgenden Fragen sind nur zu beantworten, wenn Sie Daten per App an uns übermitteln würden.

11. Was wäre Ihnen bei einer solchen App wichtig?

	sehr wichtig	wichtig	weniger wichtig	nicht wichtig
Pseudonymisierung[1] / Datenschutz	☐	☐	☐	☐
Feedback vom Arzt / klinischen Personal aufgrund meiner Eingaben	☐	☐	☐	☐
Einfache Bedienung	☐	☐	☐	☐
Multilingual / Angebot in meiner Muttersprache	☐	☐	☐	☐

[1] Anonymisierung / Pseudonymisierung: Die **Anonymisierung** ist das Verändern personenbezogener Daten derart, dass diese Daten nicht mehr einer Person zugeordnet werden können. Bei der **Pseudonymisierung** wird der Name durch ein Pseudonym (zumeist eine mehrstellige Buchstaben- oder Zahlenkombination) ersetzt, um die Identifizierung des Betroffenen auszuschließen. Die Pseudonymisierung ermöglicht – unter Zuhilfenahme eines „Schlüssels" – die Zuordnung von Daten zu einer Person. Diese Zuordnung ist ohne diesen Schlüssel nicht oder nur schwer möglich ist, da Daten und Identifikationsmerkmale getrennt sind.

12. Welcher Art der Datenübermittlung würden Sie zustimmen? (Mehrfachauswahl möglich)

- ☐ direkt an einen Server der Klinik (in verschlüsselter Form)
- ☐ Speicherung in einer Cloud[2]
- ☐ als E-Mail Anhang
- ☐ Dateneingabe vor Ort: z.B. Eingabe von Daten mit einem Tablet der Klinik
- ☐ Damit kenne ich mich nicht aus.
- ☐ egal

13. Wie oft wären Sie bereit Dateneingaben z.B. zu (Lebensqualitäts-)Fragebögen über die App zu machen? (Mehrfachauswahl möglich)

- ☐ einmal im Monat
- ☐ alle 3 Monate
- ☐ alle 6 Monate
- ☐ jährlich
- ☐ zu den jeweiligen Nachsorgeterminen
- ☐ eigenständig

14. Wie viel Zeit würden Sie für die Dateneingaben aufwenden?

- ☐ Weniger als 5 Minuten
- ☐ 5 – 15 Min
- ☐ 15 – 30 Min
- ☐ 30 – 45 Min
- ☐ mehr

15. Fänden Sie es gut, wenn Ihr betreuender Arzt bereits vor der Nachsorgeuntersuchung die eingegebenen Daten (Nebenwirkungen, Lebensqualität, Laborwerte) vorliegen hat?

☐ Ja ☐ nein, weil ______________________________

16. Würden Sie einer Verwendung Ihrer anonymisierten oder pseudonymisierten Daten zur Verbesserung der Therapie und Auswertung für Studien zustimmen?

☐ ja ☐ nein, weil ______________________________

17. Verwenden Sie andere **Geräte** für eHealth (Health-Tracking, Gesundheitsevaluation) z. B. Fitnessarmbänder?

☐ ja ☐ nein

Wenn ja, welche Geräte: ______________________________

18. Verwenden Sie bereits eine andere **App** für eHealth (Health-Tracking, Gesundheitsevaluation)?

☐ ja ☐ nein

Wenn ja, welche Apps: ______________________________

19. Würden Sie es begrüßen, wenn Nachsorgeuntersuchungen in Zukunft durch Online-Angebote (z.B. Apps) ergänzt werden?

☐ ja ☐ nein

20. Würden Sie sich eine direkte Kontaktaufnahme bei Unregelmäßigkeiten Ihrer Eingaben (z.B. bei auffälligen Laborwerten, starken Nebenwirkungen, etc.) wünschen?

☐ ja ☐ nein

---

[2] Unter **Cloud Speicherung** versteht man das Speichern von Daten in einem entfernten Rechenzentrum, also nicht auf dem lokalen Arbeitsplatzcomputer oder Server, sondern entfernt in der (metaphorischen) Wolke (englisch: cloud).

Im Folgenden werden Zusatzfunktionen der App beschrieben.

21. Welche der folgenden Zusatzfunktionen der App würden Sie nutzen? (Mehrfachauswahl möglich)

- ☐ Visualisierung der Eingaben (Laborwerte als Tabelle, Verlauf der Nebenwirkungen)
- ☐ Terminkalender während Therapie (z. B. für Chemotherapie- oder Bestrahlungstermine)
- ☐ Erinnerung an Termine (Bestrahlungstermine, Nachsorgetermine, etc.)
- ☐ Erinnerung an das Ausfüllen von (Lebensqualitäts-)Fragebögen, aktuelle Nebenwirkungen, Gesundheitszustand
- ☐ Nachschlagewerk und Wissenswertes zu Ihrer Therapie (Pflegehinweise, Übungen, Wegepläne, etc.)

22. Wie wichtig wären Ihnen allgemein Zusatzfunktionen bei einer App zur Unterstützung Ihrer Therapie und Nachsorgezeit?

☐ sehr wichtig ☐ wichtig ☐ weniger wichtig ☐ nicht wichtig

23. Haben Sie selbst Vorschläge für Zusatzfunktionen, die den Nutzen einer derartigen App für Sie erweitern würde?

____

____

____

24. Haben Sie Kommentare zur Umfrage?

____

____

____
