# Supplementary material for: Mobile Health in Oncology: A Patient Survey About App-Assisted Cancer Care
Source: JMIR Mhealth Uhealth. 2017 Jun 14;5(6):e81. doi: 10.2196/mhealth.7689 (PMC5489709; doi:10.2196/mhealth.7689)
Supplement: Multimedia Appendix 2 [file mhealth_v5i6e81_app2.pdf]

Below possible additional functions of such an app are described.

21. Which of the following additional functions would you use? (multiple selection possible)

- ☐ Visualization of data entry (blood values in tables, development of side effects over time)
- ☐ Calendar during therapy (e. g. dates of chemotherapy or radiation, etc.)
- ☐ Date reminder (e. g. dates of radiation, follow-up dates, etc.)
- ☐ Reminder for completing questionnaires on life quality, side effects and health
- ☐ Guide and interesting facts considering your therapy (care instructions, exercises, building plans, etc.)

22. How important would you consider additional functions for supporting your therapy and follow-up time?

☐ very important ☐ important ☐ less important ☐ not important

23. Do you have suggestions for additional functions of an app which would support you in your therapy?

____

____

____

24. Do you have comments on this survey?

____

____

____

PATIENT SURVEY

Dear patient,

apps for smartphones and tablets are nowadays used on a constant basis and support people in their everyday life. Even in health care electronic/mobile devices are often applied. This is widely known as *eHealth*.

We would like to find out if we could use such apps for your benefit in our clinic. But we need your help for this! We would appreciate if you take a few minutes of your time and answer the questions below for the implementation of a mobile health app in oncology.

Please return the completed questionnaire at the registration desk or put it in the provided boxes. All data will be treated in strict confidence.

We thank you for your help!

With best wishes for a speedy recovery,

Your

Stephanie E. Combs

1. General information:

   Sex
   ☐ female
   ☐ male

   Age ____________________

   Mother tongue ____________________

2. In which department at MRI are you treated?

   ____

3. Because of which tumor disease are you treated?

   ____

4. Which therapy you receive or have received in the past? (multiple selection possible)

   ☐ Radiotherapy ☐ Chemotherapy ☐ Surgery

5. Which mobile device do you use? (multiple selection possible)

   ☐ Smartphone ☐ Tablet ☐ PC/Notebook ☐ None

6. Which operating system does your smartphone/tablet use? (multiple selection possible)

   ☐ Android ☐ iOS (Apple) ☐ WindowsMobile ☐ BlackberryOS

   ☐ Others: ______________________________ ☐ I don't know

7. How would you rate your experience in handling mobile devices?

   ☐ very good ☐ good ☐ intermediate ☐ poor

8. Do you use apps in your everyday life?

☐ yes ☐ no

I use the following apps: ____

9. Would you use a mobile health app and transfer data about your health condition via a secure line?

☐ yes ☐ no

9.1. If **yes**, which data or information would you transfer? (multiple selection possible)

☐ Test results (blood values e. g. PSA values)
☐ Data on treatment satisfaction
☐ Data on life quality and well-being
☐ Examination results (e. g. CT/MRT)
☐ Data on subjective side effects associated with the therapy

☐ other: ____

9.2. If **no**, why would you not transfer data? (multiple selection possible)

☐ I don't have a capable device.
☐ I don't know about the technique.
☐ I don't know what happens with the data.
☐ I don't know where my data is stored.
☐ I am afraid of a non-secure line for transferring data.
☐ Reasons of data protection (I don't want to transfer non-anonymized[1] data.)
☐ I only want personal contact to a doctor.
☐ I don't think it's reasonable.

☐ other reasons: ____

10. Would you use an app if you would be compensated, e.g. your health insurance would offer a bonus for the use?

☐ yes ☐ no

You only need to answer the following questions if you would agree on transferring data to us.

11. Which facts on the app would you consider as important?

	very important	important	less important	not important
Pseudonymization[1] / Data protection	☐	☐	☐	☐
Feedback by doctor based on my input	☐	☐	☐	☐
Easy handling	☐	☐	☐	☐
Multilingual / Service in my mother tongue	☐	☐	☐	☐

[1] Anonymization / Pseudononymization: **Anonymization** is the process of changing personal data so that it can't be associated to the person who previously entered the data. On the other hand, **pseudonymization** is the procedure in which the person's name is replaced with a pseudonym (usually a combination of numbers and letters). An identification of the person who previously entered the data by non-authorized personal is not possible, but with the help of a „key" which is only known by authorized persons the identification is possible. Without this "key" the identification is impossible because data and identifiers are stored separately.

12. Which way of data transmission would you prefer? (multiple selection possible)

- ☐ Directly to a clinics server (via a secure line)
- ☐ Storage in a Cloud[2]
- ☐ As an e-mail attachment
- ☐ On-site data entry: e. g. entering data in a clinics‘ tablet
- ☐ I don’t know about those matters.
- ☐ I don’t care.

13. How often would you be willing to enter data via an app? (multiple selection possible)

- ☐ Once a months
- ☐ Every 3 months
- ☐ Every 6 months
- ☐ yearly
- ☐ at follow-up appointments
- ☐ independently

14. How much time are you willing to take for data entry?

- ☐ Less than 5 minutes
- ☐ 5 – 15 minutes
- ☐ 15 – 30 minutes
- ☐ 30 – 45 minutes
- ☐ more

15. Would you prefer if your treating doctor would have access to your data (side effects, life quality, test results) at your follow-up appointments?

☐ yes ☐ no, because ____

16. Would you agree on the usage of your anonymized or pseudonomyized data for improving therapy and clinical studies?

☐ yes ☐ no, because ____

17. Do you use any **devices** for eHealth (health tracking) e.g. fitness bands?

☐ yes ☐ no

If yes, which devices: ____

18. Do you already use **apps** for eHealth (health tracking)?

☐ Yes ☐ No

If yes, which apps: ____

19. Would you appreciate, if follow-up appointments are complemented with online services (e.g. apps) in the future?

☐ yes ☐ no

20. Would you wish to be contacted directly by a doctor if you take irregular data entries (e.g. abnormal blood test, heavy side effects, etc.)

☐ yes ☐ no

---

[2] **Cloud storage** is the storage of data in a distant data center. The data is stored in a (metaphorical) cloud and not on local hard discs of desktop computers or on servers.
